# Infection of an Insect Vector with a Bacterial Plant Pathogen Increases Its Propensity for Dispersal

**DOI:** 10.1371/journal.pone.0129373

**Published:** 2015-06-17

**Authors:** Xavier Martini, Mark Hoffmann, Monique R. Coy, Lukasz L. Stelinski, Kirsten S. Pelz-Stelinski

**Affiliations:** Citrus Research and Education Center, Entomology and Nematology Department, University of Florida, Lake Alfred, Florida, United Sates of America; Volcani Center, ISRAEL

## Abstract

The spread of vector-transmitted pathogens relies on complex interactions between host, vector and pathogen. In sessile plant pathosystems, the spread of a pathogen highly depends on the movement and mobility of the vector. However, questions remain as to whether and how pathogen-induced vector manipulations may affect the spread of a plant pathogen. Here we report for the first time that infection with a bacterial plant pathogen increases the probability of vector dispersal, and that such movement of vectors is likely manipulated by a bacterial plant pathogen. We investigated how *Candidatus* Liberibacter asiaticus (*C*Las) affects dispersal behavior, flight capacity, and the sexual attraction of its vector, the Asian citrus psyllid (*Diaphorina citri* Kuwayama). *C*Las is the putative causal agent of huanglongbing (HLB), which is a disease that threatens the viability of commercial citrus production worldwide. When *D*. *citri* developed on *C*Las-infected plants, short distance dispersal of male *D*. *citri* was greater compared to counterparts reared on uninfected plants. Flight by *C*Las-infected *D*. *citri* was initiated earlier and long flight events were more common than by uninfected psyllids, as measured by a flight mill apparatus. Additionally, *C*Las titers were higher among psyllids that performed long flights than psyllid that performed short flights. Finally, attractiveness of female *D*. *citri* that developed on infected plants to male conspecifics increased proportionally with increasing *C*Las bacterial titers measured within female psyllids. Our study indicates that the phytopathogen, *C*Las, may manipulate movement and mate selection behavior of their vectors, which is a possible evolved mechanism to promote their own spread. These results have global implications for both current HLB models of disease spread and control strategies.

## Introduction

The spread of vector-transmitted pathogens relies on complex interactions between host, vector and pathogen [1, 2]. Pathogen-mediated mechanisms that manipulate the interactions of vector-host systems are suggested to have long-reaching effects on ecosystem dynamics and structure [3–6]. Most of our understanding concerning the impact of vector-borne parasites on vector behavior comes from studies focused on pathogens of medical and veterinary importance, and much less is known about plant pathosystems [7–10]. In contrast to animal pathosystems, plants are mainly sessile hosts and vectors are often the most mobile element in a plant pathosystem [11, 12].

Currently, understanding movement patterns of insect vectors and the effects of parasites or pathogens on these patterns is limited. Generally, movement dynamics of a parasitizing insect can be simplified to a choice between staying on a host or leaving it to search for a new host [13, 14]. Plant viruses can influence acceptance of hosts by insect vectors. For example, Ingwell et al [11] showed that aphids, *Rhopalosiphum padi*, infected with the Barley Yellow Dwarf Virus (BYDV) preferentially settled on uninfected wheat plants, while uninfected aphids preferred BYDV-infected plants. According to Ingwell et al. [11], acquisition of a plant virus has a direct effect on host plant selection by *R*. *padi* and impacts vector behavior in a manner that promotes the spread of the virus. Because the spread of a vector-transmitted plant pathogen is closely tied to vector dispersal [15–17], movement of a vector may be manipulated by pathogens to increase their own spread.

Sap feeding insects are good models to address this question given that several are efficient vectors of plant pathogens [18, 19] and many are of significant agricultural importance because they transmit phytopathogens that cause significant crop loss. The Asian citrus psyllid (*Diaphorina citri* Kuwayama, Hemiptera: Liviidae) is a phloem-feeding insect on citrus and related species of the *Rutaceae* family that transmits bacterial plant pathogens within the ‘*Candidatus* Liberibacter’ genera. In North America, *D*. *citri* transmits *Candidatius* Liberibacter asiaticus’ (*C*Las), which is associated with huanglongbing (HLB). HLB is the most devastating disease of citriculture globally [20, 21]. We investigated the *Citrus–D*. *citri*–*C*Las pathosystem to determine whether and how a bacterial plant pathogen impacts the movement of its insect vector. *Diaphorina citri* is a hemimetabolous insect that develops on newly emerging leaves referred to as ‘flush’ [22, 23]. Whereas the *C*Las pathogen is mainly acquired by flightless, immature *D*. *citri* nymphs [24], *C*Las is spread between infected and uninfected trees by winged and mobile adults. Adult *D*. *citri* are capable of 300 m flights on average with a maximum flight capability of 2.4 km without wind assistance [25, 26]. Martini et al. [27] captured *D*. *citri* in a Florida forest 2 km from the closest citrus orchard. Similarly, protein-marked adult *D*. *citri* were observed 2 km away from the initial marking area in a mark-capture investigation [28]. Other studies confirmed that adult *D*. *citri* frequently migrate from one field to another [29, 30]. Dispersal and flight behavior of adult *D*. *citri* are related to chemical stimuli that attract or repel adult *D*. *citri*. Male adults are attracted to the odors of female adults [31–33]; however, female *D*. *citri* are more attracted to plant odors than to male *D*. *citri* [32]. In addition, females are repelled by high densities of conspecific females and prefer uninfested flush over flush previously infested by conspecifics [33]. These findings indicate that female *D*. *citri* are the drivers of plant colonization. It has been demonstrated that *C*Las manipulates the odors released by its host plant according to the ‘deceptive host phenotype hypothesis’ [34]. This elicits initial attraction of *D*. *citri* to *C*Las-infected plants from which they subsequently disperse following potential acquisition of *C*las, because of the lower nutritional quality of infected, as compared with uninfected plants [13]. However, questions still remain as to whether *C*Las can manipulate vector behavior directly in addition to affecting release of host plant volatiles.

In the present study, we addressed the hypothesis that *C*Las manipulates behavior of its vector to enhance its own spread. We found that acquisition of *C*Las by *D*. *citri* affected the movement patterns of infected psyllids by increasing short and long-range dispersal. We also found that acquisition of the pathogen rendered female *D*. *citri* more attractive to males as compared with uninfected counterparts. In both cases, manipulation of vector behavior was positively correlated with the titer of the pathogen in the vector. Our investigation indicates that movement patterns of insect vectors can be manipulated by a bacterial plant pathogen. This is the first report suggesting manipulation of insect movement behavior due to infection with a bacterial plant pathogen.

## Results

### Short distance dispersal behavior of *D*. *citri* is influenced by intraspecific density and *C*Las exposure

**Fig 1:**
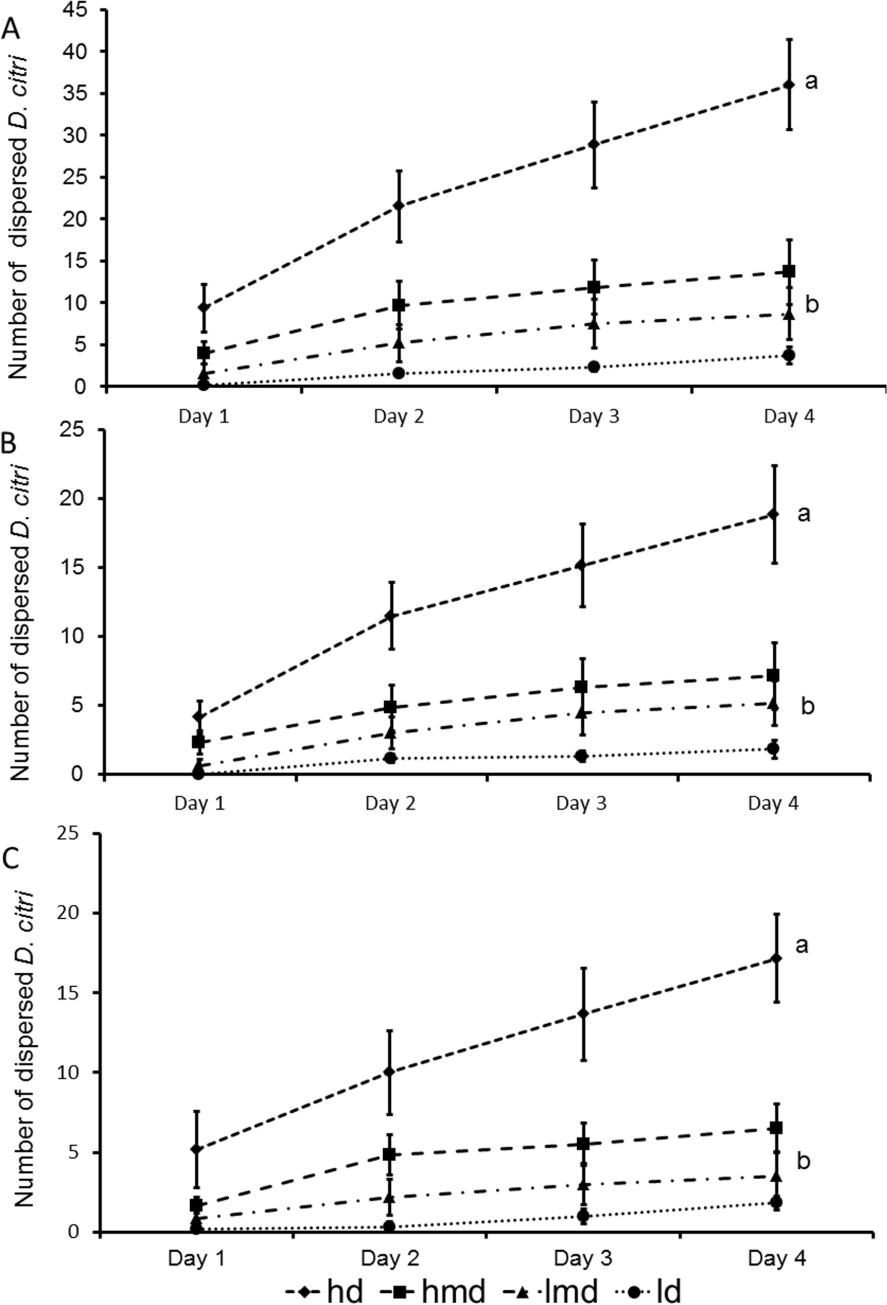
Density-dependent dispersal behavior of *D*. *citri*. The dispersal of *D*. *citri* increased with increasing density. (A) Dispersal of both male and female *D*. *citri*. a = Krukal-Wallis ANOVA: p < 0.001; (B) dispersal of female *D*. *citri*; a = Krukal-Wallis ANOVA: p < 0.001; (C) dispersal of male *D*. *citri*. a = Krukal-Wallis ANOVA: p < 0.001. hd: high density (175 individuals per plant); hmd: high medium density (125 ind. p. p.); lmd: low medium density (75 ind. p. p.); ld: low density (25 ind. p. p.). Mean cumulative numbers are shown (+- SE) per day of dispersed individuals. No significant difference was found between the number of dispersed *D*. *citri* among the ld, lmd and hmd variants. However, the dispersal of *D*. *citri* in the hd variant was significantly higher than in all other variants on all dispersal days. Male and female dispersal exhibited the same pattern.

We examined the impact of *C*Las exposure on the short distance dispersal behavior of *D*. *citri*. One key driver for dispersal is population density. In theory, high population densities at feeding sites should lead to higher food and mating competition and increase the pressure to escape and colonize sites with lower population densities [35, 36]. For *D*. *citri*, the impact of population densities on dispersal behavior is not yet known, even though repellency between females in a density-dependent manner has been demonstrated [33]. Consequently, to validate our experimental design, we first investigated the effects of increasing population densities on the dispersal of adult *D*. *citri*. Among the four densities of adults investigated, we observed a significant shift in the number of dispersing individuals between the two highest density levels examined (125 and 175 individuals per plant; Kruskal Wallis test *H* = 46.07, *P* < 0.001; Fig 1*A*). We observed the same dispersion pattern in male (Kruskal-Wallis test *H* = 45.39, *P* < 0.001; Fig 1*C*) and female individuals (Fig 1*B*). We examined the impact of *C*Las exposure on the dispersal behavior of *D*. *citri* with the highest population density (175 adult *D*. *citri* per plant), because psyllids showed highest dispersal at this density.

**Fig 2:**
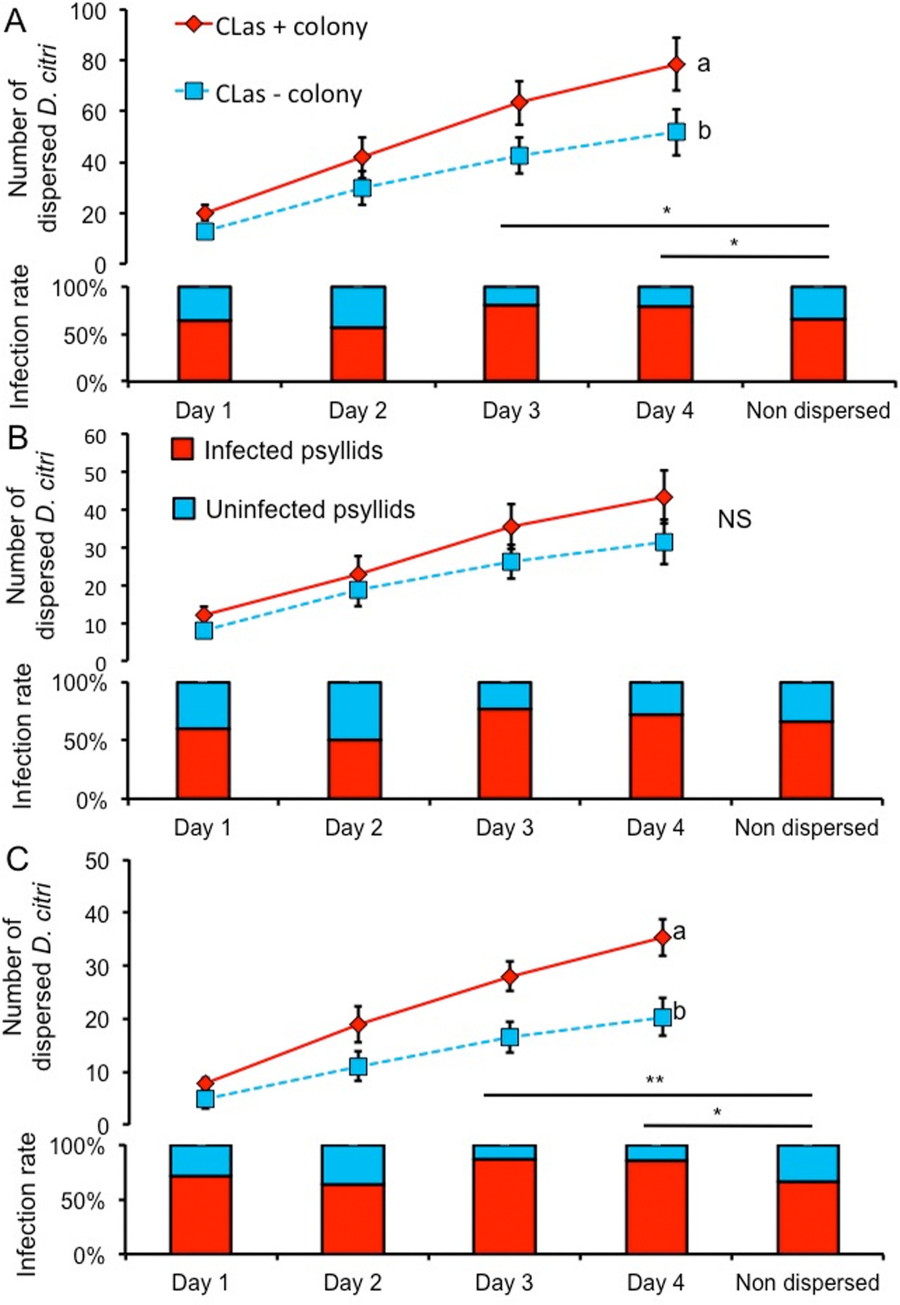
Dispersal behavior of *D*. *citri* depending of *C*Las exposure and infection. Dispersal of psyllids according to gender: (A) male and female; (B) female; and (C), male. Cumulative dispersal of psyllids is indicated by line graphs. Bars indicate the infection status of dispersing or non-dispersing *C*Las-exposed *D*. *citri* over four days. (A) Cumulative dispersal of *D*. *citri*. *C*Las-infected *D*. *citri* dispersed more than uninfected *D*. *citri* on day 3 (*P* = 0.011, GLM) and day 4 (*P* = 0.041, GLM), but not on days 1 and 2. (B) Cumulative dispersal of female *D*. *citri*. There were no significant differences in the dispersal and infection rates of *C*Las-exposed *D*. *citri* females. (C). Cumulative dispersal as compared between *C*Las-infected and uninfected male *D*. *citri* (*P* = 0.007, Kruskal-Wallis).The bottom graph shows the infection rates of dispersed *C*Las-exposed *D*. *citri* males. *C*Las-infected *D*. *citri* males dispersed more than uninfected *D*. *citri* males on day 3 (*P* = 0.003, GLM) and day 4 (*P* = 0.001, GLM), but not on days 1 and 2.

Exposure of *D*. *citri* to *C*Las-infected plants during nymphal development resulted in higher numbers of dispersing adults as compared with uninfected controls (Kruskal-Wallis test *H* = 3.92, *P* = 0.048; Fig 2*A*). Interestingly, only male adults exhibited increased dispersal behavior after exposure to *C*Las-infected plants as juveniles as compared with controls (Kruskal-Wallis test *H* = 7.25, *P* = 0.007; Fig 2*C*). Although females exhibited a slightly greater tendency to disperse if they were exposed to *C*Las-infected plants as juveniles, no statistically significant differences were observed between exposed and unexposed females (Kruskal-Wallis test *H* = 2.362, *P* = 0.124; Fig 2*B*). Among those insects exposed to *C*Las-infected plants during their development, an average of 65% harbored *C*Las as an adult (Fig 2*A*). Whereas the infection rate was the same among the psyllids that dispersed, as compared with the psyllids that did not disperse, during the initial two days of the experiment, the infection rate was significantly higher in dispersing *D*. *citri* at day 3 (GLM with binomial distribution χ^2^ = 6.41, df = 1; *P* = 0.011; Fig 2*A*) and day 4 (GLM with binomial distribution χ^2^ = 4.195, df = 1; *P* = 0.041; Fig 2*A*) as compared with psyllids that did not disperse. This suggests that infection of *D*. *citri* with *C*Las increases their propensity for dispersal as compared with uninfected counterparts. These patterns were only observed in adult male *D*. *citri* at day 3 (GLM with binomial distribution χ^2^ = 8.64, df = 1; *P* = 0.003; Fig 2*C*) and day 4 (GLM with binomial distribution χ^2^ = 6.26, df = 1; *P* = 0.012; Fig 2*C*), but not in females (Fig 2*B*). Our results indicate gender-specific effects of *C*Las on dispersal of *D*. *citri*, resulting in significant higher dispersal rates of adult males as compared to uninfected counterparts, although a similar trend was observed in females.

### Effects of *C*Las infection and *C*Las exposure on flight capacity of *D*. *citri*

**Fig 3:**
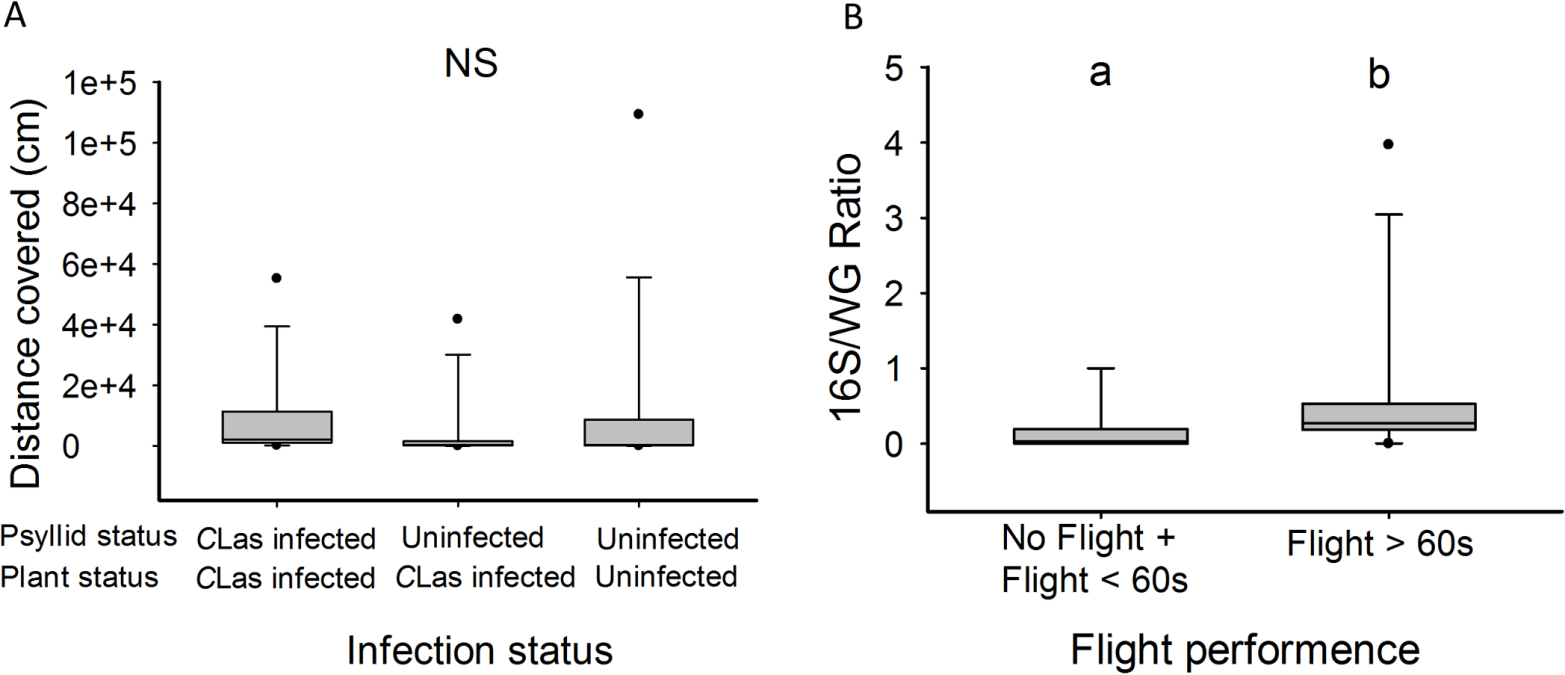
Flight performance of *D*. *citri* depending of *C*Las exposure and infection. (A) Distance covered by psyllids on the flight mill depending on their infection status and the infection status of the rearing plants, as determined by qPCR. (B) Ratio between *16S* and *Wg* genes depending on flight performance of psyllids on the flight mill. The ratio of 16S/WG increased proportionally with *C*Las DNA quantified in individual *D*. *citri*. The 16S/WG was significantly higher in infected *D*. *citri* that performed long flights (> 60s) than in infected *D*. *citri* that did not fly or performed short flights (< 60s) (*P* = 0.033, Kruskal-Wallis). A line within each boxplot indicates the median for each treatment and symbols above each boxplot indicate outliers.

To investigate if *C*Las exposure and infection affects dispersal of *D*. *citri*, we assessed the flight capacity of *C*Las-exposed and unexposed adult *D*. *citri* on a flight mill apparatus that has been described in detail previously [26]. The number of *D*. *citri* that did not fly did not differ among the various treatments tested (GLM with binomial distribution χ^2^ = 3.2858, df = 2; *P* = 0.193). Among those *D*. *citri* that did fly, significantly more performed longer flights (> 60s) following exposure to and acquisition of *C*Las than uninfected counterparts (GLM with binomial distribution χ^2^ = 6.00, df = 2; *P* = 0.049, Table 1). However, the overall distance flown by *D*. *citri* did not differ statistically between these treatments at the α < 0.05 level (Kruskal-Wallis test *H* = 5.86, *P* = 0.054; Fig 3*A*).

**Table 1:** Percentage of *D*. *citri* adults tested on a flight mill that did not fly (‘non flyers’); performed only short-duration flights (‘short flyer’, < 60 s); or performed long-duration flights (‘long flyer’, > 60 s). Flight capabilities of *D*. *citri* were compared by considering *C*Las infection status of psyllids and host plants. Also included is the maximum duration of flight recorded for each category.

Host plant^1^	Psyllid^1^	Short flyer (%)	Long flyer (%)	N^2^	Sex (% female)	Non Flyer (%)	Flight initiation (s)^3^	Speed (cm/s)^4^
*C*Las^+^	*C*Las^-^	58.33	41.67^a,b^	23	60.86	47.83^a^	151.50 ± 43.15^b^	12.08 ± 1.42^b^
*C*Las^+^	*C*Las^+^	21.42	78.57^a^	19	68.47	26.32^a^	46.85 ± 27.73^a^	16.77 ± 1.45^a^
*C*Las^-^	*C*Las^-^	61.11	38.89^b^	24	79.17	25.00^a^	133.94 ± 33.85^b^	16.74 ± 1.31^a^

Different letters following percentages indicate significant differences (α<0.05) among cells within the same column.

^1^ *C*Las^+^ and *C*Las^-^ refer to plants or psyllids that have tested positive (^+^) or negative (^-^) for the pathogen in qPCR analyses, respectively.

^2^Number of psyllids tested on the flight mill for each category.

^3^ Average time (s) needed for psyllid to initiate a flight (non-flyers excluded).

^4^ Average velocity that *D*. *citri* flew for each category (non-flyers excluded)

Although we did not find a linear correlation between flight duration and *C*Las infection ratio (16S/Wg) (*F*_*1*,*18*_ = 0.07, *P* = 0.778), the *16*S/*Wg* ratio was significantly higher in infected *D*. *citri* which performed flights > 60s than in infected counterparts which did not fly or performed flights < 60s (Kruskal-Wallis test *H* = 3.9307, *P* = 0.047; Fig 3*B*). These results suggest a direct increase in the probability of performing a long flight due to the acquisition of the *C*Las pathogen by the vector. Interestingly, *D*, *citri* that acquired the pathogen (as determined by qPCR) initiated flight sooner than uninfected counterparts (Kruskal-Wallis test *H =* 6.846, *P* = 0.033*;* Table 1). Flight speed was significantly lower for exposed, but uninfected *D*. *citri* than exposed and infected or unexposed/uninfected *D*. *citri* (ANOVA *F*_*2*,*39*_ = 3.37, *P* = 0.045).

There was no gender specific difference in flight capacity; however, male wings were significantly shorter than female wings (2.37 ± 0.018 mm versus 2.287 ± 0.026 mm, respectively; ANOVA *F*_*1*,*52*_ = 7.8; *P* = 0.007). There was no significant difference in wing length between exposed and infected, exposed and uninfected or unexposed and uninfected individuals (ANOVA *F*_*2*,*52*_ = 0.503, *P* = 0.608). Also, there was no correlation between sex and infection status (ANOVA *F*_*2*,*52*_ = 2.04, *P* = 0.140).

### Response of *D*. *citri* males to odors of *C*Las-exposed females

**Fig 4:**
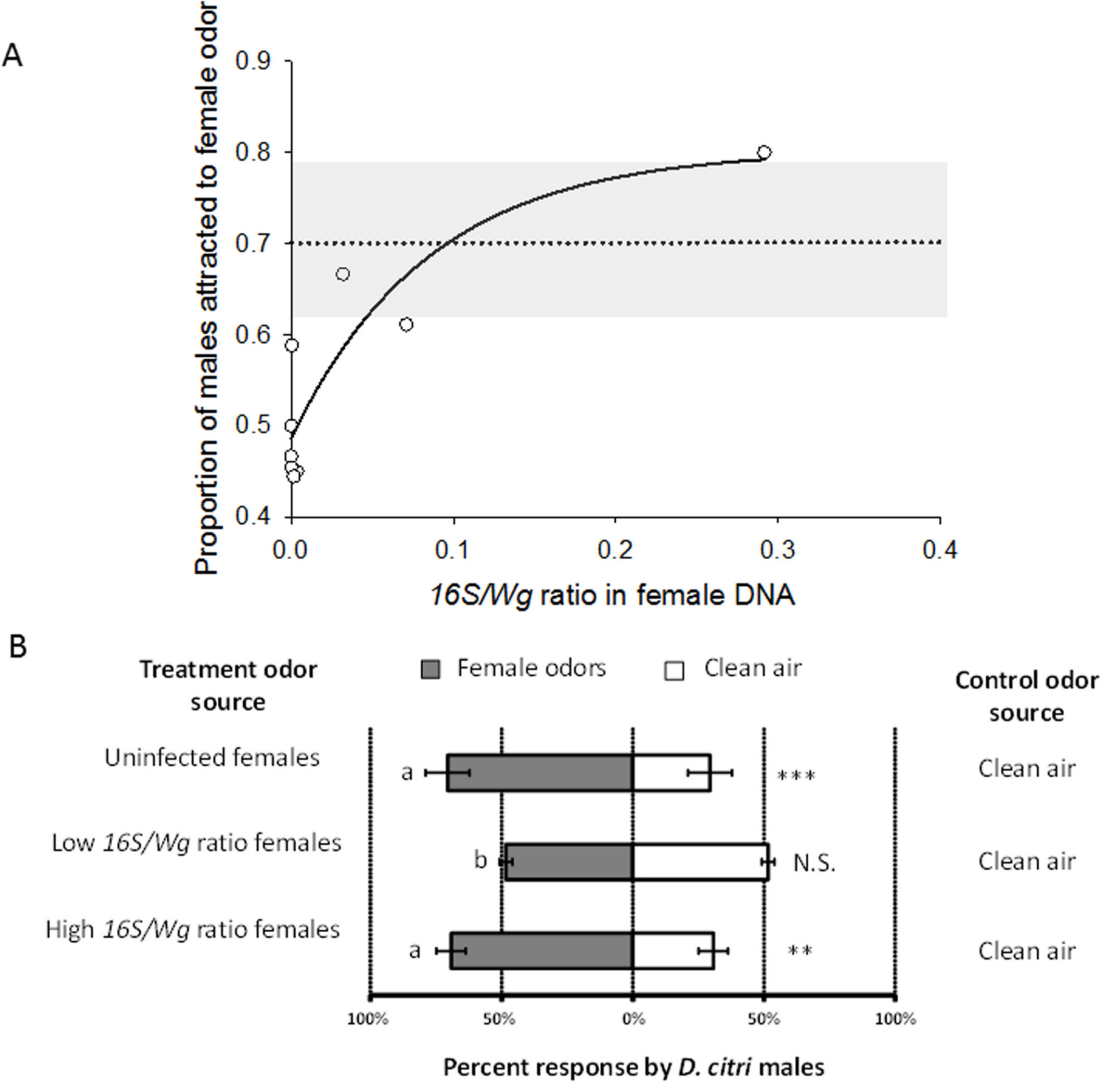
Behavioral response of *D*. *citri* males to headspace volatiles from conspecific females exposed to *C*Las. (A) Proportion of males attracted to female odor and (B) percentage of males responding to clean air verses female odors representing each infection status. (A) Male responses are plotted against the average ratio between *16S* and *Wg* genes of the ten females of each replicate placed in the treatment arm. Females developed on *C*Las-infected citrus plants and the *16S*/*Wg* ratio indicates the amount of *C*Las DNA found per individual *D*. *citri*. Each circle indicates the proportion of males that chose the olfactometer arm with conspecific female odor for each replicate. Each replicate consisted of 20 males resulting in the test of 180 males. The dotted line and the grey area represent the average response (± SEM) of males when exposed to uninfected (control) female odors versus clean air. Control females were reared on uninfected citrus plants and were free of *C*Las. The regression equation was: *y* = 0.48 + 0.34(1 − *e* ^*−6*.*76x*^); R^2^ = 0.87, F_2,6_ = 20.31; P = 0.002. (B) Asterisks indicate significant attraction of *D*. *citri* males to female odor (**: < 0.01; ***: <0.001) compared to clean air. Different letters indicate significant differences in the proportion of male psyllids responding to the female odors among infection status treatments.

In a T-maze olfactometer choice test (Analytical Research System, Gainesville, FL) [37], we compared the response of male *D*. *citri* to odors of *C*Las-exposed versus unexposed females. The response of males toward the odor of *C*Las-exposed females versus clean air was significantly correlated with *C*Las infection ratio (*1*6S/*Wg*) of the female placed in the treatment arm (Fig 4A) (Non-linear regression equation: *y* = 0.48 + 0.34(1-*e* ^-6.76*x*^); R^2^ = 0.87, *F*_*2*,*6*_ = 20.31, *P* = 0.002.). Significantly more males entered an arm containing 10 unexposed females compared with an arm with clean air (χ^2^ = 7.69, df = 1,P = 0.005; Fig 4A). Females exposed to *C*Las-infected plants, but harboring low titers of the pathogen (16S/Wg < 0.01), were not significantly more attractive to males (χ^2^ = 0.1481, df = 1, *P* = 0.70) compared with clean air. Conversely, females exposed to *C*Las-infected plants and harboring high titers of the pathogen (16S/Wg > 0.01) were consistently more attractive to males compared with clean air (χ^2^ = 7.0784, df = 1, *P* = 0.008). The proportion of male psyllids responding to female odors differed significantly among the odor treatments (ANOVA: *F*_*2*,*10*_ = 10.93, df = 2, *P* = 0.003). Females from uninfected plants and females from *C*Las-infected plants with high titers of the pathogen (16S/Wg > 0.01) were more attractive to males than females exposed to *C*Las-infected plants with low titers of the pathogen (16S/Wg < 0.01) (*P* = 0.006, *P* = 0.011, respectively; Fig 4B).

## Discussion

Our results indicate both direct and indirect effects of a bacterial plant pathogen on the movement patterns of its vector. *C*Las appeared to manipulate inclination for dispersal, flight capacity, and sexual attraction of *D*. *citri*. Ultimately, these effects increased the movement of those vectors that harbored the pathogen as compared with uninfected counterparts, which may promote spread of *C*Las by *D*. *citri* following pathogen acquisition. To our knowledge, this is the first description of direct changes to insect behavior mediated by a bacterial pathogen in a vector-plant-pathogen system. Our results are consistent with other findings of coevolved mechanisms between pathogens, hosts, and vectors, in which pathogens manipulate hosts in a manner predicted to favor transmission and spread of the pathogens [7, 11, 38]. Our results complement and extend previous findings regarding plant pathogen-induced behavioral manipulations of vectors, which focused mainly on indirect changes of host choice behavior and feeding mediated by changes in host (plant) chemistry [13, 39, 40]. Our observation of pathogen-induced increases in flight initiation closely parallel findings of pathogen infection modifying insect host locomotor activity to facilitate their spread, such as the pathogen-induced host climbing behavior of Lepidoptera larvae and increased activity of *Aedes aegypti* [41, 42].

Theoretical and empirical studies have demonstrated that spread of plant-pathogen mediated diseases is often associated with the frequency of vector dispersal [12, 15–17]. Notably, the increase of short-distance dispersal by the vector is likely to favor multiple inoculations of the same host at different locations, whereas an increase in long-distance dispersal may favor the spread of the pathogen to new hosts. *C*Las infection increased the probability of both short and long distance flights by *D*. *citri*, as compared with uninfected controls; however, the duration and the velocity of the recorded long flights were not affected following acquisition of the pathogen (as determined by qPCR). This may be due to physiological boundaries in the amount of energetic reserves or in the output of flight muscles of *D*. *citri* irrespective of infection status. Therefore, possible manipulation of long and short-range dispersal of the vector by the pathogen may be behavioral and limited by physiological capacity.

The impact of pathogen infection on sexual behavior of *D*. *citri* is less obvious regarding its possible consequences for pathogen spread. First, the increase of female attractiveness to males following *C*las infection may result in a higher rate of pathogen transmission during copulation [43]. Even if the rate of transmission of *C*Las through mating is low [43], it is likely that the probability of sexual transmission would increase with the infection level of the conspecific partner. Therefore, sex-related transmission during mating among *D*. *citri* is likely to increase proportionally with the bacterial titer found within psyllids. Second, assuming that female *D*. *citri* are more likely to exploit new food sources and oviposition sites than males, and that males follow the volatile signature of females [31–33], lesser attraction of males to uninfected females or those harboring low titers of *C*Las, than to those harboring high titers, may increase spread of males to unexplored feeding sites. In addition, females with higher titers of *C*Las were more attractive to males and performed longer duration flights at a greater frequency than females with lowered titers. Therefore, highly infected females appear more likely to explore new hosts. Also, given the greater attractiveness of these females to males, there may be a greater chance not only to infect a new host, but also to establish a new colony of *D*. *citri* that will become vectors of *C*Las following acquisition from infected plants as compared with uninfected, plant tissue. These hypotheses warrant further testing and mathematical modeling to further explore the epidemiology of HLB spread.

An intriguing aspect of our results is that female sexual attraction and, to a lower extent, flight capacity, were both reduced when psyllids developed on infected plants and were also not infected with *C*Las or harbored low titers following acquisition. The lesser attractiveness of female *D*. *citri*, lower flight speed, and lower inclination for initiating flight of psyllids that were either uninfected or which contained low titers of *C*Las, as compared with those that contained high titers of *C*Las, may be indicative of a difference in plant quality between infected and uninfected plants. For instance, the phloem of *C*Las-infected citrus is characterized by higher starch content [44, 45] and a potential reduction in nutritional value to *D*. *citri* [13, 46, 47]. This decrease in nutritional quality may be the result of induced plant defense following pathogen infection [48] or it may be correlated with general decline of the diseased and dying host. It has been demonstrated that feeding of *D*. *citri* on *C*Las-infected plants is lower than on uninfected counterparts [13]. Also, an investigation of *D*. *citri* feeding on *C*Las-infected plants has demonstrated that the durations spent feeding from the phloem is reduced on infected, as compared with uninfected, plants as measured by the electro-penetration graph technique [49]. These results suggest that *C*Las-infected plants are a lower quality food resource for *D*. *citri* and may impact their fitness. Those *D*. *citri* that have lesser capability of obtaining necessary nutrients from infected plants of declining quality (and perhaps lower fitness than those capable of exploiting infected plants) may suffer subsequent compounding fitness consequences in terms of dispersal and/or mating behavior. Alternatively, it is possible that acquisition of *C*Las by *D*. *citri* in some manner compensates for the presumably lower nutritional value of their diet on infected, as compared with uninfected, plants by enhancing dispersal and mating behavior.

Our results indicate that a bacterial plant pathogen (*C*Las) affects the behavior of its vector (*D*. *citri*) following acquisition. Male *D*. *citri* behavior is directly affected by acquisition of this pathogen in a titer-dependent matter so as to increase inclination for initiating dispersal behavior as compared with uninfected controls. Furthermore, female *D*. *citri* become more attractive to males following acquisition of *C*Las as compared with uninfected counterparts. Both of these behavioral manipulations may have evolved to promote spread of pathogen. Further behavioral investigation in the field, as well as, epidemiological modeling is warranted to determine how these potential behavioral modifications may influence the spread of HLB or other important phytopathogens that limit food crop production.

## Materials and Methods

### Maintenance of insects, pathogen, and host plants

Uninfected adult *D*. *citri* used in bioassays were obtained from a laboratory colony continuously reared at the University of Florida Citrus Research and Education Center (Lake Alfred, USA). The colony was established in 2000 from field populations in Polk Co, FL, U.S.A. (28.0′N, 81.9′W) prior to the discovery of HLB in the State. Insects were maintained on uninfected ‘Valencia’ sour orange (*Citrus sinensis* L.) or on curry leaf (*Murraya koenigii*) at 27±1°C, 63±2% RH, and a 14:10 L:D photoperiod. Plants were obtained as potted seedlings from a local nursery. All plants were tested for the presence of *C*Las by quantitative real-time polymerase chain reaction (qPCR) analysis [24] before they were used in the rearing process. Furthermore, all plants in the colony undergo frequent testing to ensure no *C*Las infection was present. Monthly testing of randomly sampled *D*. *citri* nymphs and adults was conducted using qPCR (described below) to confirm that the insects did not harbor *C*Las.

*Diaphornia citri* were infected with *Candidatus* Liberibacter asiaticus by rearing a colony on *C*Las-infected and HLB symptomatic *C*. *sinensis* plants. The colony was maintained in a secure quarantine facility at the above-mentioned conditions. The initial parental generation of the *C*Las-exposed colony originated from the uninfected colony described above. Monthly sampling conducted concurrently with our experiments indicated that between 25 to 55% of *D*. *citri* individuals obtained from the infected colony harbored *C*Las within the detectable range of the qPCR assay employed (20 adult *D*. *citri* individuals per monthly sampling). *C*Las in plants was maintained by graft-inoculating *C*Las uninfected ‘Valencia’ *C*. *sinensis* with *C*Las-infected vegetative plant tissue (budwood) collected from commercial citrus groves in Immokalee, FL (Collier Co.). Leaf tissue of successfully grafted plants was tested for the presence of *C*Las by qPCR 6 months after grafting. Only *C*Las-infected plants were used for rearing the *D*. *citri C*Las-infected colony. Uninfected plants were maintained in separate, secure greenhouses to minimize the chance of cross contamination

It is known that *D*. *citri* reach their sexual maturity and their maximal flight capacity four days following emergence [25, 50]. Given that *D*. *citri* have an approximate lifespan of 40 days at 25°C following adult emergence [51], we elected to perform behavioral assays using psyllids aged between 5 and 15 days after adult emergence. To obtain psyllid between 5 and 15 days old, four *M*. *koenegii* were infested with approximately 100 unsexed *D*. *citri* adults. After one week, all of these adults were removed. Thereafter, the cage was inspected daily and the adults that emerged were transferred to a separate plants free of *D*. *citri*. The day of the transfer was noted and these psyllids were used 5 to 15 days following their transfer.

### DNA isolation and qPCR detection of CLas

DNA from *D*. *citri* samples was isolated using the Blood and Tissue DNA Extraction Kit (Qiagen, Valencia CA). The concentration and purity of the extracted DNA was measured spectrophotometrically using a Nano Drop 2000 (Thermo Fisher Scientific, Waltham, MA). The presence of *C*Las was assessed by the detection of the *16S* rDNA gene by quantitative polymerase chain reaction (qPCR) [52]. In brief, a multiplex *Taq*Man (Applied Biosystems, Foster City, CA) assay targeting the *16S* rDNA of *C*Las and the *D*. *citri wingless* gene (*Wg*) was applied to detect *C*Las in *D*. *citri* samples. Standard curves for *16S* and *Wg* assessments were generated by serial dilutions of PstI linearized plasmids. Duplicate amplifications of each sample were conducted, using *Wg* primers (WgF: 5’-GCTCTCAAAGATCGGTTTGACGG-3’; WgR: 5’GCTGCCACGAACGTTACCTTC-3’), *Wg* probe (5’-JOE-TTACTGACCATCACTCTGGACGC-3BHQ2-3'), *C*Las 16S primers (LasF: 5’-TCGAGCGCGTATGCGAATAC-3’; LasR: 5’-GCGTTATCCCGTAGAAAAAGGTAG-3’) and the CLas *16S* probe (5’-56FAM-AGACGGGTGAGTAACGCG-3BHQ2-3'). Quantitiative PCR settings were: (1) 2 min at 50°C, (2) 10 min at 95°C and (3) 40 cycles with 15 sec at 95°C and 60 sec at 60°C (data collection). The reactions were processed in 7500 Fast Real-Time PCR System (Applied Biosystems), using Micro Amp Fast Optical 96-Well Reaction Plates (Applied Biosystems) and Micro Amp Optical Adhesive Film (Applied Biosystems). Gene copy numbers of 16S (*C*Las) and Wg (*D*. *citri*) were calculated based on plasmid generated standard curves. The amount of *C*Las was determined according to the assessed gene copy numbers by calculating the 16S/Wg ratio. The concentrations of reagents, plasmid construction, and reaction protocol are described in Coy et al. [52].

### Short distance dispersal behavior of *D*. *citri* is influenced by intraspecific density and *C*Las exposure

Adult *D*. *citri* individuals (4–7 days old) were released in a cage on a potted *C*. *sinensis* plant (= settling plant). After two days, four similar *C*. *sinensis* (cv. Valencia) plants were placed into the cage surrounding the settling plant at a distance of 15 cm (= dispersal plants). Settling and dispersal plants were of the same size and in the same conditions (S1 Table). Insects that were not located on the settling plant were removed before adding the dispersal plants to the experiment. The dispersal of released *D*. *citri* was assessed daily for four days after the experiment was initiated. Dispersal of *D*. *citri* was measured by counting and collecting individual insects that moved from the settling plants to the dispersal plants. After counting, the sex of each collected adult was assessed using a microscope. All dispersal experiments were repeated six times.

### Impact of population density on dispersal behavior of *D*. *citri*

The aim of this experiment was to assess the impact of population density on dispersal behavior of *D*. *citri*. We used this experiment as a positive control, as we expected a possible density dependent increase of *D*. *citri* dispersal for the various density treatments of adult *D*. *citri* (5–7 days old). We investigated the propensity of *D*. *citri* movement at various densities of *D*. *citri* placed on settling plants: 25, 75, 125 and 175 psyllids per settling plant. Each population was released in a separate cage on a settling plant, following the procedure described above. To assess density dependent differences in dispersion of male and female *D*. *citri*, a Kruskal Wallis ANOVA (*P* = 0.05) was performed. Group significances were analyzed after a Bonferroni correction with Mann Whitney U Test (*P* = 0.05). The statistics were performed with Statistica 12 (Stat Soft).

### Impact of *C*Las on dispersal behavior of *D*. *citri*

The objective of this experiment was to determine the impact of *C*Las acquisition by *D*. *citri* on their subsequent dispersal behavior. For this purpose, 175 *D*. *citri* adults (5–7 days old) were reared on either *C*Las-infected or uninfected plants. Thereafter, psyllids were released on settling plants in separate cages with surrounding dispersal plants, as described above. All collected *D*. *citri* that dispersed, as well as 20 of the insects remaining on the settling plant by end of the 4^th^ counting day, were placed into 100% ethanol for subsequent assessment of *C*Las titer via qPCR (described above). Before DNA extraction, the sex of each insect was assessed using a microscope. Infection rates of the *C*Las-exposed or unexposed colony were analyzed by Kruskal–Wallis ANOVAs to assess possible differences in *D*. *citri* dispersal as influenced by infection status. To assess whether *C*Las acquisition by *D*. *citri* affected psyllid dispersal, the infection rate of psyllids from the *C*Las-exposed colony that dispersed was compared with the infection rate of psyllids from the *C*Las-exposed colony that did not disperse using a general linear model (GLM) with a binomial distribution.

### Effects of *C*Las infection and *C*Las exposure on flight capacity of *D*. *citri*

We used a flight mill described in [26]. Briefly, the flight mill was composed of a horizontal axis consisting of an optic fiber (13 cm) fixed to a metal fiber pivot. The pivot was positioned vertically under a magnet. To maintain the metal fiber in a vertical position, a second magnet was positioned 1 cm below at the opposite end. At each extremity of the horizontal optic fiber, two 1 cm optic fiber pieces were glued vertically. The pronotum of psyllids was glued to the tip of one of the two small optic fiber pieces with non-toxic washable glue (Elmer’s products, Columbus, OH). Five to 15 day old *D*. *citri* adults were placed on an ice pack covered with filter paper. We only selected psyllids with green/blue abdominal color, as it was found that this color morph was able to perform long duration of flights [26]. While immobilized on the ice pack, psyllids were attached to the optic fiber with water soluble glue. Then, the optic fiber was glued to the horizontal axis of the flight mill and the experiment data recording was initiated. If a psyllid did not fly during the first 10 minutes, it was removed and referred as to a ‘non flyer’. If a psyllid flew, the experiment was terminated 5 minutes after the psyllid ceased to fly. Flight duration and number of rotations on the flight mill were recorded. The distance flown by each psyllid was calculated by multiplying the number of rotations by 13π. After each flight ended, the sex of the psyllid tested was determined. Additionally, we measured wing length of a sub-sample of *D*. *citri* tested on the flight mill. These were measured with a micro-ruler inserted into the lens of a dissecting microscope (Wild M3C, Leica, Wetzlar, Germany) and confirmed with the image analyzing software, ImageJ [53].

To investigate whether the infection status of the psyllid or of the host plant may change the flight capacity of *D*. *citri*, psyllids were reared on either *C*Las-infected or uninfected Valencia *C*. *sinensis* plants. Psyllids were subsequently submitted to qPCR to determine their infection status. After emergence, adult psyllids that developed on both infected and uninfected plants were transferred onto a uninfected ‘Valencia’ seedling 5 to 7 days before being tested on the flight mill. Within this timeframe on an uninfected plant, a *C*Las-infected psyllid will not become uninfected, as determined by qPCR [24]. After being tested on the flight mill, all the psyllids that developed on a *C*Las-infected plant were tested by qPCR for *C*Las infection status.

The psyllids used on the flight mill were classified in three categories: psyllids that did not fly during the experiment were categorized as ‘non flyers’; those that flew less than 60 s were categorized as ‘short flyers’; and those that flew 60 s or more were categorized as ‘long flyers’ [26]. It has been shown previously that the flight capacity of *D*. *citri* on a flight mill does not differ between the sexes [25, 26]; consequently, results from males and females were pooled. We first used a GLM with binomial distribution to compare the proportion of non-flyers, as function of infection status of the psyllid and of the host plant. Subsequently, the proportion of long flyers within the psyllids that flew was compared as function of infection status of the psyllid and of the host plant with a GLM with binomial distribution. After excluding non-flyers, we also compared the time to initiate flight and the distance covered by flight using a Kruskal-Wallis rank sum test and the flight speed with a one-way ANOVA. Within the group of *D*. *citri* that were infected, the relationship between flight duration and *C*Las infection ratio (*1*6S/*Wg*) was assessed using a linear regression. Also, the *C*Las infection ratio (*1*6S/*Wg*) was compared between long flyers versus short and non-flyers with a Kruskal-Wallis rank sum test. Wing length data were analyzed using a two-way ANOVA with sex and infection status as fixed variables.

### Response of *D*. *citri* males to odors of *C*Las infected females

A two-port divided T-maze olfactometer (Analytical Research System, Gainesville, FL) was used to evaluate the behavioral response of *D*. *citri*. The olfactometer consisted of a 30 cm long glass tube with 3.5 cm internal diameter that was bifurcated into two equal halves with a polytetrafluoroethylene (PTFE) strip forming a T-maze [37]. Each half served as an arm of the olfactometer enabling the *D*. *citri* to make a choice between two potential odor fields. To ensure chemical free ambient air supply, both arms of the olfactometer received charcoal purified and humidified air from a custom made air delivery system (ARS, Gainesville, FL). A constant airflow of 0.1 L. min^-1^ was maintained through both arms of the olfactometer. The olfactometer was positioned vertically under a fluorescent 23 W light source (FLE23HT3/3/SW, GE Lighting, Cleveland, OH) mounted within a 1.0×0.6×0.6 m fiberboard box for uniform light diffusion. The measured light intensity was approximately 600 lux above the T-maze. Male *D*. *citri* adults were released individually at the base of the olfactometer and allowed 300 s to exhibit a behavioral response. A positive response was recorded when a male psyllid moved from the base and entered 1 cm into either arm of the olfactometer. Those psyllids that did not leave the base of the olfactometer were designated as non-responders. Odor sources were randomly assigned to one arm of the olfactometer at the beginning of each bioassay and the T-maze was rotated every five psyllids to eliminate positional bias. In addition, prior to odor testing, *D*. *citri* adult males were exposed to clean air vs. clean air in the olfactometer to verify the absence of positional bias. The olfactometer arms were connected to upstream odor sources consisting of glass tubes containing 10 female *D*. *citri* reared on either *C*Las^-^ or *C*Las^+^ plants. This bioassay has been previously shown to be efficient in determining attraction of male *D*. *citri* to conspecific female odors [31–33].

Subsequently, all females used as attractant sources were submitted to qPCR for detection of *C*Las (see above). All assays were conducted between 9:00 and 14:00 hrs. All psyllids used for these bioassays were of the green-blue color morph [26] and previously mated, given that preliminary earlier experiments have shown that males are more responsive to female odors when both males and females had previously mated [31, 54]. For each treatment, there were four replicates of 20 to 24 male *D citri*. Chi-square tests on pooled results from four replicates were used to compare the number of *D*. *citri* entering the treatment or control arm of the olfactometer. The response of male *D*. *citri* to odors of females that acquired the *C*Las pathogen, as compared with those that were not infected, was compared with a linear mixed-model with binomial distribution. The response of males among treatments was compared with ANOVA, followed by the Holm-Sidak test.

## Supporting Information

S1 Table Properties of settling and dispersal plants (means ± SE). Dispersal and settling plants were of the same stem height, abundance of flush [cm], and number of flush per plant (Mann-Whitney U Test).
(DOCX)
