# Supplementary material for: Infection of an Insect Vector with a Bacterial Plant Pathogen Increases Its Propensity for Dispersal
Source: PLoS One. 2015 Jun 17;10(6):e0129373. doi: 10.1371/journal.pone.0129373 (PMC4471203; doi:10.1371/journal.pone.0129373)
Supplement: S1 Table — Dispersal and settling plants were of the same stem height, abundance of flush [cm], and number of flush per plant (Mann-Whitney U Test). (DOCX) [file pone.0129373.s001.docx]

**Table S1:** **Properties of settling and dispersal plants (means ± SE). Dispersal and settling plants were of the same stem height, abundance of flush [cm], and number of flush per plant (Mann-Whitney U Test).**

| Plant | Stem [cm] | Flush [cm] | Nr. of flush |
| --- | --- | --- | --- |
| Settling plant | 23.38 ± 0.94^ns^ | 10.35 ± 1.96^ns^ | 2 ± 0.32^ns^ |
| Dispersion plant | 25.01 ± 0.22^ns^ | 7.98 ± 0.74^ns^ | 2.15 ± 0.14^ns^ |

^ns^ not significant (MWU Test, P = 0.05)
